# Assessment on the Effects of Glycerol Monolaurate on the Antibacterial and Beta-Lactamase *bla*_TEM_ and *bla*_SHV_ Gene Expression in Multidrug-Resistant *Klebsiella pneumoniae*

**DOI:** 10.21315/tlsr2026.37.3.7

**Published:** 2026-10-15

**Authors:** Esther-Jia-Yee Koh, Choy-Hoong Chew, Kai-Jen Leong, Yi-Hui Cheong, Quok-Cheong Choo

**Affiliations:** 1 Department of Allied Health Sciences, Faculty of Science, Universiti Tunku Abdul Rahman (UTAR), 31900 Kampar, Perak, Malaysia; 2 Department of Biological Sciences, Faculty of Science, Universiti Tunku Abdul Rahman (UTAR), 31900 Kampar, Perak, Malaysia

**Keywords:** Glycerol Monolaurate, *Klebsiella pneumoniae*, *bla*_TEM_, *bla*_SHV_, Multidrug-Resistant, Monolaurin, Klebsiella pneumoniae, bla_TEM_, bla_SHV_, Perintang Pelbagai Dadah

## Abstract

The alarming rise of antimicrobial resistance (AMR) has driven researchers to explore alternatives that can replace antimicrobial agents. Natural antimicrobials are considered promising due to their safety, sustainability and abundance in the environment. Among the various natural antimicrobials being explored, glycerol monolaurate (GML) has encouraged the investigation of its potential antibacterial properties. This study aimed to determine the antibacterial activity of GML against multidrug-resistant (MDR) Gram-negative *Klebsiella pneumoniae* and to evaluate their gene expression activity on both *bla*_SHV_ and *bla*_TEM_ genes. The broth microdilution method, with slight modification, was used to determine the minimum inhibitory concentration (MIC) and minimum bactericidal concentration (MBC) of GML, while the gene expression analysis was performed using RT-qPCR. In this study, no MIC or MBC was observed in all tested GML concentrations, indicating a lack of direct antibacterial activity against *Klebsiella pneumoniae*. However, transcriptional analysis demonstrated a significant downregulation of the *bla*_TEM_ gene, but an insignificant downregulation of the *bla*_SHV_ gene. These findings suggest that GML may reduce the resistance mechanism at the transcriptional level by modulating the expression of the beta-lactamase gene, despite lacking a direct bactericidal effect. This highlights the potential of GML as a gene expression modulator and adjuvant candidate in combination therapy, rather than as a direct antimicrobial agent.

## Abstrak

Kebangkitan isu rintangan antimikrob (AMR) yang membimbangkan telah mendorong para penyelidik untuk meneroka alternatif yang boleh menggantikan agen antimikrob. Antimikrob semula jadi dianggap berpotensi disebabkan kemampanan, keselamatan dan kewujudannya yang meluas dalam persekitaran. Antara pelbagai antimikro semula jadi yang telah dikaji, monolaurat gliserol, atau monolaurin (GML) telah menggalakkan penyelidikan lanjut berkenaan potensi antibakteria.. Oleh itu, kajian ini tertumpu kepada penentuan potensi antibakteria GML terhadap Klebsiella pneumoniae yang tahan terhadap pelbagai dadah (MDR) serta menilai aktiviti ekspresi gen bla_TEM_ dan bla_SHV_. Kaedah mikrodilusi kaldu, dengan pengubahsuaian telah digunakan untuk menentukan kepekatan perencatan minimum (MIC) dan kepekatan bakterisid minimum (MBC) bagi GML, manakala analisis ekspresi gen dijalankan menggunakan RT-qPCR. Dalam kajian ini, tiada MIC atau MBC diperhatikan pada semua kepekatan GML yang diuji, menunjukkan ketiadaan aktiviti antibakteria terhadap Klebsiella pneumoniae. Namun begitu, analisis transkripsi menunjukkan penurunan yang signifikan dalam ekspresi gen bla_TEM_, manakala gen bla_SHV_ kekal tidak berubah. Penemuan ini mencadangkan bahawa GML boleh mengurangkan mekanisma rintangan pada peringkat transkripsi dengan memodulasi ekspresi gen beta-laktamase walaupun tanpa kesan bakterisid. Hal ini menyerlahkan potensi GML sebagai pengawal selia ekspresi gen dan calon adjuvan dalam rawatan kombinasi dan bukannya sebagai agen antimikrob secara langsung.

**HIGHLIGHTS**

- No MIC or MBC observed in glycerol monolaurate (GML)-treated MDR *Klebsiella pneumoniae*.
- The expression of *bla*_TEM_ gene but not *bla*_SHV_ was significantly downregulated following GML treatment.
- GML may serve as a potential adjuvant candidate in antibacterial combination therapy.

## INTRODUCTION

With the rising trend of antibiotic resistance in healthcare settings caused mainly by the inappropriate use of antibiotics, researchers have started to shift their attention to natural products that are safe, widely available and possess promising antimicrobial properties. In Malaysia, our tropical climate supports the extensive cultivation of coconut and palm oil trees, making the country one of the world’s leading palm oil producers and exporters. These plantations produce oils rich in medium-chain fatty acids such as lauric acid (LA), which can be converted into a more bioactive compound, glycerol monolaurate (GML), through esterification with glycerol. Therefore, it is worth exploring the bioactive compounds derived from palm kernel oil (PKO) or virgin coconut oil (VCO) for their antibacterial properties.

Lauric acid is a medium-chain fatty acid with the chemical formula C_12_H_24_O_2_ and a molecular weight of 200.32 g/mol. According to Rahman *et al*. (2022), LA can be found abundantly in VCO (~54%) and PKO (~50%). However, LA has limited solubility in water, reducing the antibacterial effectiveness. Therefore, LA can be esterified with glycerol to produce a derivative known as GML, which has better solubility. GML is an ester with the chemical formula C_15_H_30_O_4_ and a molecular weight of 274.40 g/mol. Due to the additional glycerol group, it dissolves better in the aqueous solutions, which allows a greater antibacterial activity. Both LA and GML are generally recognised as safe (GRAS) compounds verified by the United States Food and Drug Administration (FDA), where LA can be used as a flavour enhancer, lubricant, and surface-active agent, and GML can be used as an emulsifier in cosmetics (Food and Drug Administration 2025).

According to the Ministry of Health Malaysia and Ministry of Agriculture and Food Security (2022), the incidence of extended-spectrum beta-lactamases (ESBL) producing *Klebsiella pneumoniae* increased from 2019 to 2021. Beta-lactamase is an enzyme capable of breaking down the beta-lactam rings present in beta-lactam antibiotics, particularly the third and fourth generations of cephalosporins and monobactams, leading to ineffective antibiotic treatments (Then 2007; Husna *et al*. 2023). These ESBL genes are predominantly plasmid-encoded, which can cause horizontal spread between bacterial strains. As a result, ESBL-producing *K. pneumoniae* harbouring the *bla*_SHV_ and *bla*_TEM_ genes are often resistant to antibiotics such as cephalosporins and penicillins, as beta-lactamases can cleave the beta-lactam ring of the antibiotics. Consequently, these antibiotics cannot bind to the penicillin-binding proteins, and no inhibition takes place in the bacterial cell wall synthesis (Patel & Preuss 2024; CDC 2024).

The evaluation of antibacterial effects of natural products should not rely solely on their impact on bacterial growth but should also consider the molecular level perspective. Growth inhibition might be less prominent, but the expression of resistance genes might be reduced, potentially restoring susceptibility towards antibiotics. As a derivative, GML has been reported to exert more substantial antibacterial effect compared to LA (Schlievert & Peterson 2012). Therefore, it is worth evaluating GML in terms of bacterial growth inhibition and the gene expression which plays a significant role.

## MATERIALS AND METHODS

### Bacterial Strains and Culture Conditions

The bacterial strains involved in this study were *Klebsiella pneumoniae* ATCC 13883 and a clinical strain of *K. pneumoniae* H6 which was collected from Hospital Raja Permaisuri Bainun, Ipoh. Bacterial strains were maintained as glycerol stocks and stored at −80°C in Luria-Bertani (LB) broth supplemented with 25% (v/v) glycerol. The bacterial strains were routinely subcultured on Tryptic Soy Agar (TSA) and incubated at 37°C for 18 to 24 h before use.

### Antimicrobial Susceptibility Test (AST)

The Kirby-Bauer disk diffusion method was used to test the antimicrobial susceptibility of *K. pneumoniae* strains according to the protocols suggested by American Society for Microbiology (Hudzicki 2009). *K. pneumoniae* strains were tested against beta-lactam and non-beta-lactam antibiotics such as ampicillin (10 μg), amoxicillin/clavulanic acid (30 μg), cefotaxime (30 μg), ceftazidime (30 μg), cefuroxime (30 μg) and ciprofloxacin (10 μg). The inhibition zones were measured and classified as sensitive (S), intermediate (I), and resistant (R) by referring to the Liofilchem antibiotic disk interpretative criteria and quality control (Liofilchem 2025). Bacterial isolate that demonstrated non-susceptibility to at least one agent in three or more categories of antimicrobial is defined as MDR (Magiorakos *et al*. 2012).

### Detection of Antibiotic Resistance Genes

Total DNA was extracted using the heat-boiling method (Kor *et al*. 2013). Briefly, overnight cultures were lysed by heating at 95°C–100°C for 5 min. The lysate was then immediately transferred to ice for 2 min and centrifuged at 11,200 x*g* for 2 min. Then, the supernatant containing the crude genomic DNA was transferred to a new microcentrifuge tube and stored at −20°C until use. The purity of extracted DNA was assessed using NanoDrop^TM^ 1000 spectrophotometer at A260/280 and electrophoresis on 1% (w/v) agarose gel.

Next, the target resistance genes (*bla*_TEM_ and *bla*_SHV_) were amplified using duplex polymerase chain reaction (PCR) using gene-specific primers to verify their presence in the bacteria. Each 20 μL reaction included 1 X Green GoTaq® Flexi Buffer, two millimole magnesium chloride (MgCl_2_) solution, 0.5 μM primers, 0.2 mM PCR nucleotide mix, 1.25 U DNA polymerase and 150 μg/mL template DNA. The primer sequences and expected amplicon sizes are listed in Table 1. PCR was performed with initial denaturation at 95°C for 15 min followed by 30 PCR cycles with denaturation at 95°C for 30 s, annealing at 55°C for 30 s, and extension at 72°C for 40 min, with a final extension at 72°C for 5 min (Perez-Bou *et al*. 2024). The PCR products were then assessed with 1% (w/v) agarose gel electrophoresis at 90 V for 45 min. ChemiDoc^TM^ XRS+ with Image Lab^TM^ was then used to visualise and capture the gel images.

### Determination of Minimum Inhibitory Concentration (MIC) and Minimum Bactericidal Concentration (MBC)

GML (purity 95%–100%) was purchased from Chem Soln. GML powder of 0.1 g was initially dissolved in 1 mL of 99% (v/v) absolute, undenatured ethanol to obtain a stock solution of 100 mg/mL. The solutions were then stepwise diluted with sterile distilled water to obtain 1 mg/mL working solutions with a final ethanol concentration of 1% (v/v). For MIC and MBC determination, the 1 mg/mL working solution was serially diluted in 1% (v/v) ethanol to obtain final concentrations of 350, 300, 250, 200, 150, 100 and 50 μg/mL, while maintaining the ethanol concentration at 1% (v/v).

The MIC of GML against *K. pneumoniae* was determined using the broth microdilution method according to the European Committee on Antimicrobial Susceptibility Testing (EUCAST) (2024) guidelines with slight modifications. A single bacterial colony was introduced to LB broth and incubated for 16 h–18 h at 37°C, 200 rpm in a shaking incubator. After the overnight incubation, one millilitre of the overnight culture was transferred to 10 mL of LB broth and cultured until it reached the log phase (OD_600_ ~0.4–0.6). The bacterial suspension was then adjusted to 0.5 McFarland standard (~1 × 10^8^ CFU/mL) using fresh Mueller-Hinton broth (MHB), followed by subsequent dilution to achieve a final inoculum of ~1 × 10^6^ CFU/mL. Then, 100 μL of prepared GML with a concentration ranging from 0 μg/mL–350 μg/mL was dispensed into each well of a 96-well plate, followed by 100 μL of the prepared bacterial inoculum. This resulted in a final bacterial inoculum of ~5 × 10^5^ CFU/mL per well (Garcia-Ruiz & Ryadnov 2022; Kadeřábková *et al*. 2024). The plates were incubated at 37°C for 24 h. After incubation, the wells were then observed for turbidity. The minimum inhibitory concentration (MIC) was determined when a clear well was observed, which indicated no visible bacterial growth.

For minimum bactericidal concentration (MBC) determination, 100 μL of bacterial suspension from the clear wells was spread onto Mueller-Hinton agar (MHA) and incubated for 24 h at 37°C to observe for bacterial growth. Plates with no visible colony growth observed were recorded as MBC, which corresponds to a ≥ 99.9% reduction in the initial bacterial inoculum.

### RNA Extraction and Gene Expression Analysis

Total RNA of bacterial cells was extracted using TRIzol^TM^ reagent according to the protocol guidelines from Thermo Fisher Scientific Inc. Extracted RNA was then stored at −80°C for long-term preservation and to avoid degradation. The concentration and purity of the extracted RNA were assessed using a NanoDrop^TM^ 1000 spectrophotometer with wavelengths at 230 nm, 260 nm and 280 nm. RNA quality was determined using the ratios A260/A280 and A260/A230. Then, the RNA integrity was confirmed using 1% (w/v) denaturing agarose gel electrophoresis. The RQ1 RNase-Free DNase (Promega) was used to remove potential DNA contamination in the extracted RNA prior to RT-qPCR.

The expression of *bla*_SHV_ and *bla*_TEM_ genes was then determined with RT-qPCR using the HiScript II One-Step qRT-PCR SYBR Green Kit. Each 20 μL reaction included 10 μL of 2× one-step SYBR Green reaction mix, 1.0 μL one-step SYBR Green enzyme mix, 0.2 μM forward primer, 0.2 μM reverse primer, 40.3 ng/μL template RNA and 7.2 μL of RNase-free double-distilled water. Amplification was performed using the CFX96^TM^ Real-Time PCR Detection System. The primer sequences and the RT-qPCR conditions are mentioned in Table 1.

The relative gene expression levels were then analysed using the 2^−ΔΔCt^ method, and the results were normalised to the housekeeping genes *rec*A and *rpo*D. All reactions were performed in triplicate to ensure the consistency of the results.

### Statistical Analysis

The GraphPad Prism software was used to analyse the data collected from the MIC assay and RT-qPCR. All data were analysed and expressed in the form of mean ± standard deviation (SD). Statistical differences among the groups were performed using one-way analysis of variance (ANOVA) or an independent t-test, with *p* < 0.05 considered statistically significant.

## RESULTS

### Antibiotic Susceptibility Test

*K. pneumoniae* ATCC 13883 showed resistance towards both ampicillin and amoxicillin/clavulanic acid, while remaining sensitive towards cefotaxime, ceftazidime, cefuroxime and ciprofloxacin. In contrast, *K. pneumoniae* H6 showed resistance towards all the antibiotics tested, including ampicillin, amoxicillin/clavulanic acid, cefotaxime, ceftazidime, cefuroxime and ciprofloxacin. This profile indicated a broader resistance spectrum in *K. pneumoniae* H6 compared to the ATCC strain. Therefore, this AST confirmed that *K. pneumoniae* H6 was multidrug-resistant, allowing subsequent molecular detection of associated resistance genes. The average inhibition zone diameters (mm) for *K. pneumoniae* are presented in Table 2.

### Duplex PCR Detection on *bla*_TEM_ and *bla_SHV_* Genes in *K. pneumoniae*

Duplex PCR was performed to confirm the presence of *bla*_TEM_ and *bla*_SHV_ genes in *K. pneumoniae*. The agarose gel image in Fig. 1 demonstrated the presence of *bla*_TEM_ and *bla*_SHV_ genes with amplicon sizes of approximately 275 bp and 104 bp, respectively. The results obtained were correlated with the results in antibiotic susceptibility tests (AST) performed earlier, where resistance to beta-lactam antibiotics corresponded with the detection of these beta-lactamase genes.

### Minimum Inhibitory Concentration (MIC) and Minimum Bactericidal Concentration (MBC) Assays

The antibacterial effect of GML against *K. pneumoniae* ATCC 13883 and H6 was limited, as no MIC or MBC were observed. As shown in Fig. 2, there was a significant reduction (*p* < 0.0001) in the relative bacterial growth percentage when *K. pneumoniae* strains were treated with 50 μg/mL of GML. However, a clear concentration-dependent inhibition was not observed, evidenced by the constant relative growth percentage as the concentration of GML increased. Moreover, there was also no significant difference detected between the two *K. pneumoniae* strains, indicating a similar response pattern towards GML treatment.

### Fold of *bla*_TEM_ and *bla*_SHV_ Gene Expression of *Klebsiella pneumoniae* H6 Under Effect of GML

The relative gene expression analysis for *bla*_TEM_ and *bla*_SHV_ genes in *K. pneumoniae* H6, as shown in Table 3, revealed differential expression of the beta-lactamase genes following treatment with 150 μg/mL of GML. The expression of the *bla*_TEM_ gene was significantly downregulated in GML-treated samples (0.44 ± 0.04) compared to the untreated control (1.00 ± 0.12), with *p* < 0.05. In comparison, the *bla*_SHV_ gene did not show a significant change between the treated (0.77 ± 0.04) and untreated (1.00 ± 0.39) samples, with *p* > 0.05. This indicated that GML selectively affects the expression of *bla*_TEM_, with *bla*_SHV_ remaining unchanged under the tested conditions.[Fig f3-tlsr-37-3-145]

## DISCUSSION

In this study, the focus was placed on the antibacterial and gene expression effects of GML on MDR *K. pneumoniae*. A previous study by Projan *et al*. (1994) reported that GML could inhibit beta-lactamase induction in *Staphylococcus aureus* by blocking the *bla*R1 signalling process, leading to beta-lactamase gene inactivation. However, there is a lack of recent research investigating the effect of GML on beta-lactamase gene expression, particularly *bla*_TEM_ and *bla*_SHV_ in Gram-negative bacteria.

Our AST results of *K. pneumoniae* H6 demonstrated resistance towards all antibiotics tested, including ampicillin, amoxicillin/clavulanic acid, cefotaxime, ceftazidime, cefuroxime and ciprofloxacin. Subsequent duplex PCR analysis confirmed the presence of both *bla*_TEM_ and *bla*_SHV_ genes, which supported the resistance profile. Although no MIC or MBC were observed following GML treatment, transcriptional analysis revealed a significant downregulation of the *bla*_TEM_ gene, while the *bla*_SHV_ gene remained unchanged. Significant downregulation of the *bla*_TEM_ gene suggested that GML possess the ability to affect the transcriptional regulation of specific beta-lactamase genes, although there was an absence of bactericidal effects.

GML demonstrated a negative antibacterial activity in this study, as neither MIC nor MBC was detected across all tested concentrations (50 μg/mL–350 μg/mL). This finding aligned with Schlievert and Peterson (2012), who also reported that *K. pneumoniae* was not susceptible towards GML treatment. In contrast, another study by Sawale *et al*. (2024) revealed that MBC were achieved against Gram-negative *Pseudomonas aeruginosa* and *Salmonella* Typhimurium at 18 mg/mL and 16 mg/mL, respectively. This discrepancy may be attributed to the different solvent systems used during the preparation of GML. In this study, the ethanol concentration was fixed at 1% (v/v) to minimise solvent-driven antibacterial effect. However, Sawale *et al*. (2024) allowed ethanol concentration to scale with GML concentration, potentially contributing to enhanced antibacterial activity due to solvent interference.

Despite the absence of MIC and MBC, there was a downregulation of *bla*_TEM_ genes found. This result suggested that GML could perform gene regulation rather than the direct killing of the bacteria. Projan *et al*. (1994) reported a similar finding where GML had successfully inhibited the induction of beta-lactamase in *S. aureus* through the interference of the *bla*R1 signal transduction pathway. Interestingly, this downregulation was only effective on the *bla*_TEM_ gene but not the *bla*_SHV_. This difference might be attributed to their differences in the regulatory mechanisms. However, there is no reported evidence to explain the transcriptional differences between the two beta-lactamase genes. The specific regulatory mechanism remains unclear, which requires additional studies to examine the underlying mechanism. The ability of GML to downregulate the *bla*_TEM_ gene might suggest that it can be a potential antibiotic adjuvant. Thus, the beta-lactam antibiotics can regain their ability to kill bacteria through the inactivation of penicillin-binding proteins and interfere with the bacterial cell wall synthesis.

There are several limitations observed in this study. Firstly, there were limited studies focused on the antibacterial effect of GML. Therefore, limited comparisons on the results can be made. Secondly, focus was only given to the Gram-negative *K. pneumoniae*, without the involvement of Gram-positive bacteria. Since there was study reported that GML possess greater inhibitory effects against Gram-positive bacteria compared to Gram-negative bacteria; therefore, including Gram-positive bacteria in future studies would allow more comprehensive comparison in both antibacterial activity and their transcription profiles (Sawale *et al*. 2024). Thirdly, beta-lactamase protein levels and enzymatic activity were not evaluated in this study. The expression of mRNA levels did not always reflect the protein abundance. Therefore, incorporating the beta-lactamase assay or protein quantification in future studies is important to assess whether GML has successfully downregulated both gene and protein.

## CONCLUSION

In conclusion, this study demonstrated that GML was insufficient to exert a bactericidal effect on *K. pneumoniae* H6. Molecular analysis further showed a selective downregulation of the *bla*_TEM_ gene, with no detectable effect on the *bla*_SHV_ gene. This finding indicated that GML may have the potential to modulate the beta-lactamase gene rather than causing direct inhibition of bacterial growth. Such gene modulation suggests that GML could act as a potential antibiotic adjuvant, assisting in the restoration of beta-lactam antibiotics’ efficacy against the resistant strains.

## Figures and Tables

**FIGURE 1:**
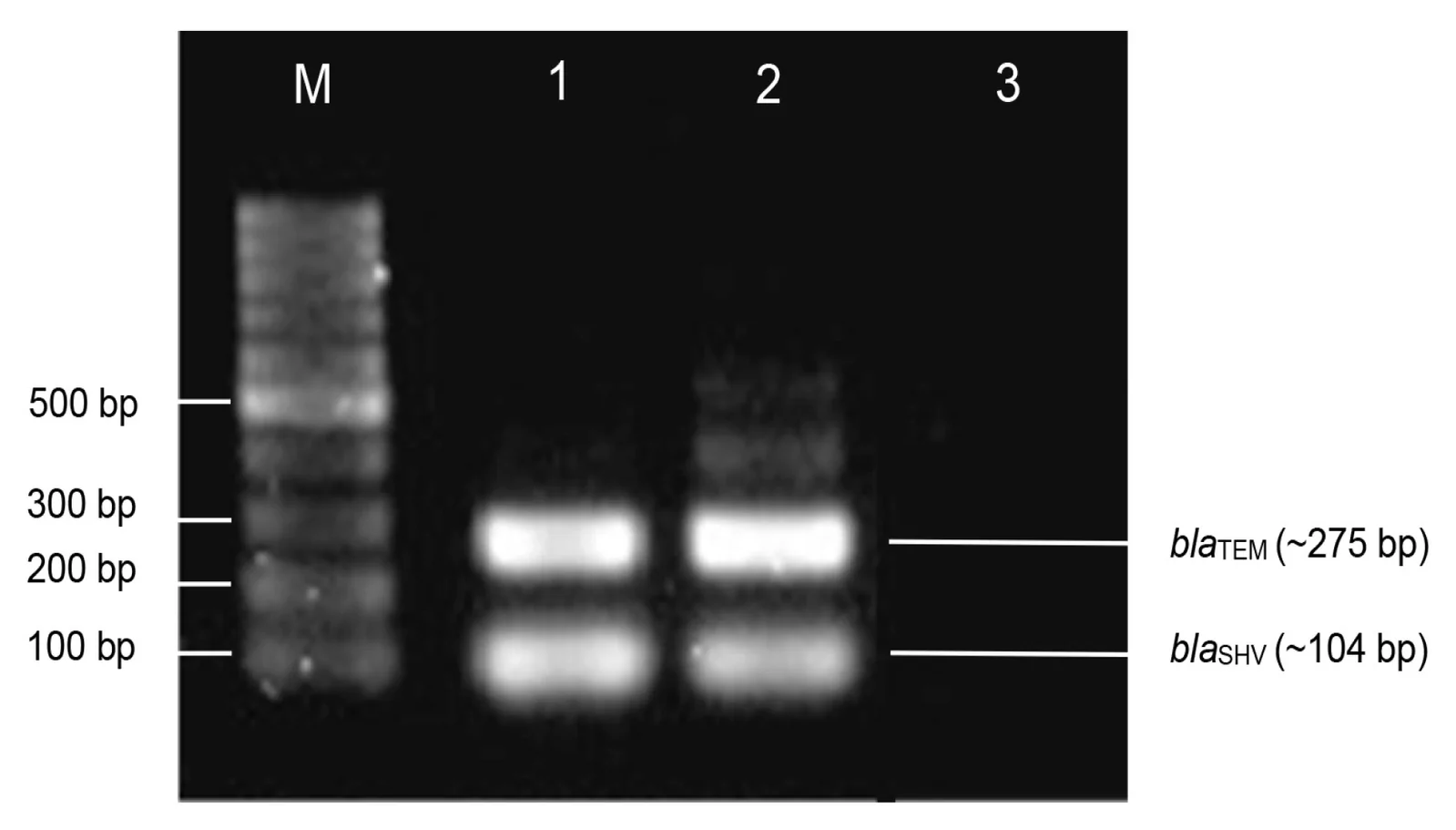
The agarose gel image shown two prominent bands – *bla*_TEM_ and *bla*_SHV_ genes after polymerase chain reaction (PCR) at 275 bp and 104 bp. Lane M: 100 bp molecular weight marker, lane 1: *Klebsiella pneumoniae* ATCC 13883, lane 2: *Klebsiella pneumoniae* H6, lane 3: sterile distilled water as negative control.

**FIGURE 2:**
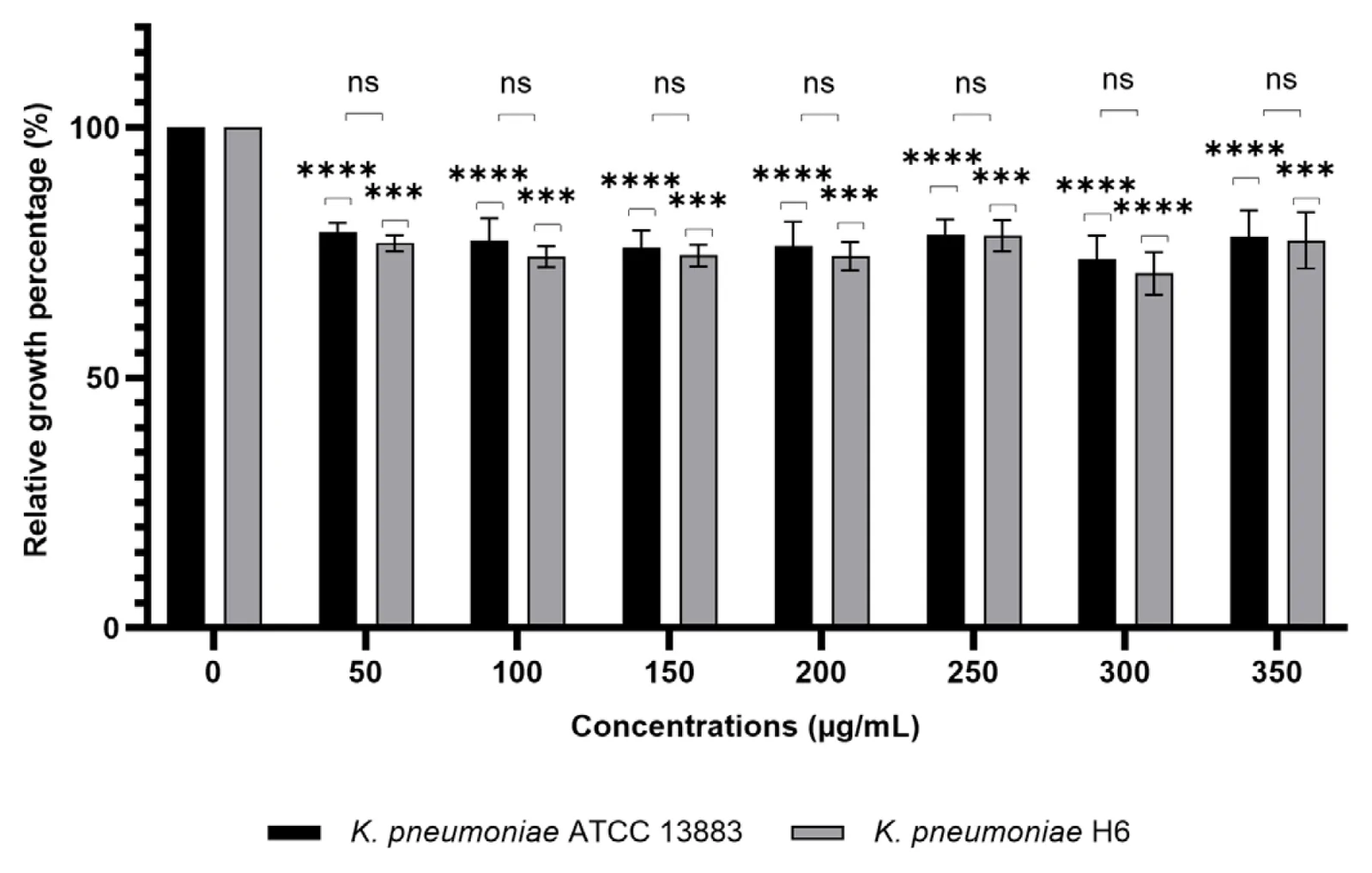
The antibacterial effect of GML against *K. pneumoniae* ATCC 13883 and H6. The graph shows the relative growth percentage of bacterial plotted against different concentrations of GML. The error bars representing mean ± standard deviation of three independent experiments. Each treatment concentration was compared to the negative control (0 μg/mL). One-way ANOVA followed by Dunnett’s post hoc test were used to determine statistical significance *p < 0.05; **p < 0.01; ***p < 0.001; ****p < 0.0001; ns = not significant, *p* > 0.05.

**FIGURE 3 f3-tlsr-37-3-145:**
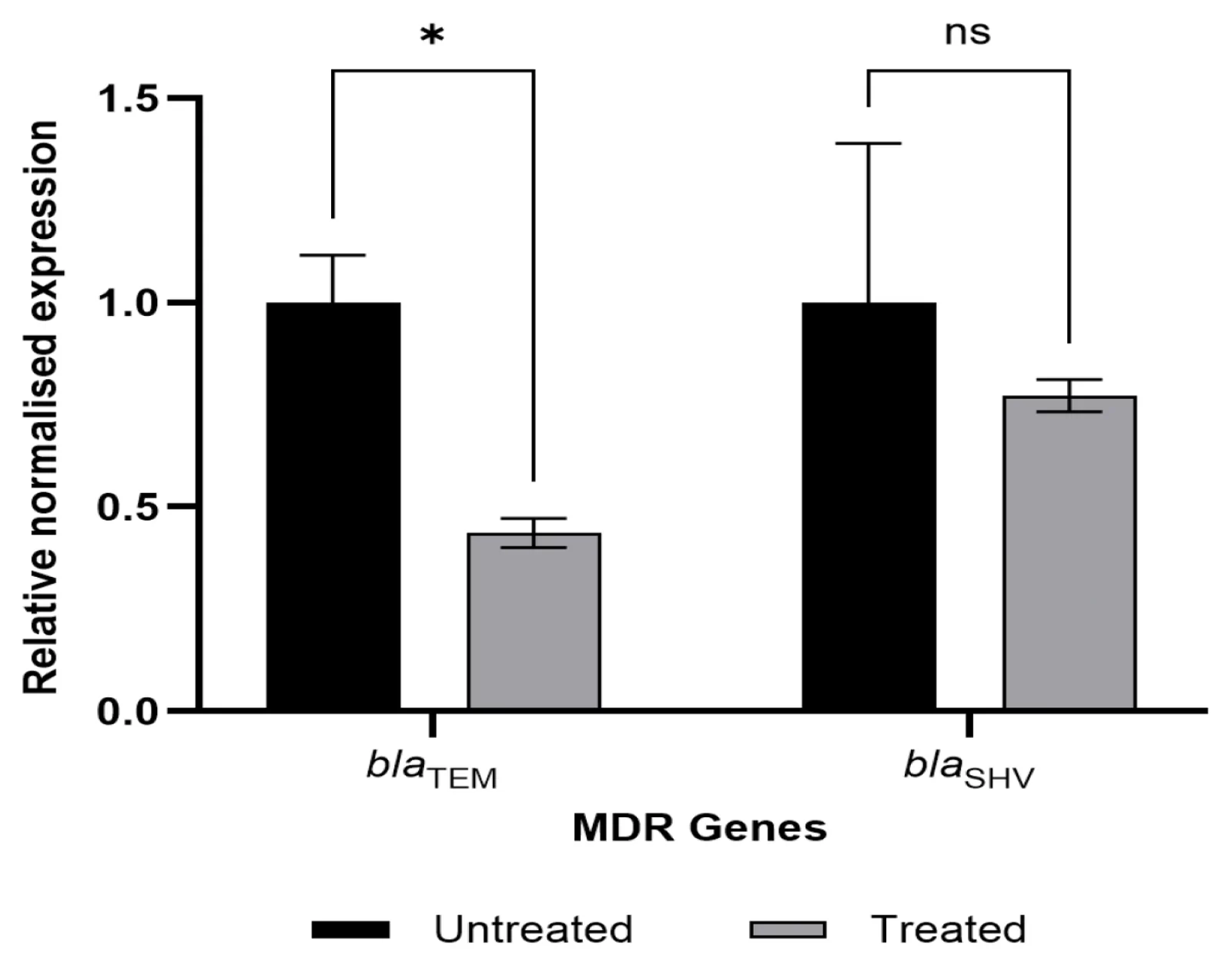
The relative normalised expression graph of *bla*_TEM_ and *bla*_SHV_ genes in *K. pneumoniae* H6 treated with 150 μg/mL GML. The error bars representing mean ± SEM (standard error mean) of three replicates. Significant differences were determined using multiple unpaired t-tests, *p < 0.05, ns= not significant, *p* > 0.05.

**TABLE 1:** Cycling conditions of RT-qPCR for *bla*_SHV,_ *bla*_TEM_, *rec*A, and *rpo*D gene amplification.

Target genes	Sequences (5′ – 3′)	Sizes (bp)	Condition	References
*bla*_SHV_	F: 5′-TCCCATGATGAGCACCTTTAAA-3′ R: 5′-TCCTGCTGGCGATAGTGGAT-3′	104	Reverse transcription at 50°C for 10 min, initial denaturation at 95°C for 30 s, followed by 36 cycles of denaturation at 95°C for 30 s, and annealing at 55°C for 30 s. A melt curve analysis at 65°C to 95°C was performed with 0.5°C increment every 5 s.	Gomes *et al*. (2018); Al-Hashimy & Al-Musawy (2020); Perez-Bou *et al*. (2024); Chen *et al*. (2025)
*bla*_TEM_	F: 5′-CGGATGGCATGACAGTAAGAG-3′ R: 5′-TTGCCGGGAAGCTAGAGTAAG-3′	275		
*rec*A	F: 5′-TTAAACAGGCCGAATTCCAG-3′ R: 5′-CCGCTTTCTCAATCAGCTTC-3′	99		
*rpo*D	F: 5′-TCCGGTGCATATGATTGAGA-3′ R: 5′-ATACGCTCAGCCAGCTCTTC-3′	105		

**TABLE 2:** Antibiotic susceptibility test (AST) for two *K. pneumoniae*.

Antibiotics	Dose (μg)	*Klebsiella pneumoniae* average inhibition zone diameters (mm)	

		ATCC 13883	H6
Ampicillin (AMP)	10	0 (R)	0 (R)
Amoxicillin/clavulanic acid (AUG)	30	10 (R)	0 (R)
Cefotaxime (CTX)	30	30 (S)	0 (R)
Ceftazidime (CAZ)	30	23 (S)	0 (R)
Cefuroxime (CXM)	30	20 (S)	0 (R)
Ciprofloxacin (CIP)	10	30 (S)	15 (R)

*Note*: R = resistant; S = sensitive.

**TABLE 3:** The relative gene expression analysis for *bla*_TEM_ and *bla*_SHV_ in *K. pneumoniae* H6 following treatment with GML (150 μg/mL). Expression values are presented as mean ± SEM relative to the untreated control. Significant differences were determined using multiple unpaired t-tests.

Target genes	Sample	Expression	ExpressionSEM	Corrected expression SEM	Mean Cq		Cq SEM *P*-value
*bla*_TEM_	GML treated (150 μg/mL)	0.43929	0.03643	0.03643	23.74	0.11169	< 0.05*
	Non-GML treated	1.00000	0.11305	0.11305	21.04	0.11379	
*bla*_SHV_	GML treated (150 μg/mL)	0.77259	0.03983	0.03983	29.62	0.00000	0.59ns
	Non-GML treated	1.00000	0.38900	0.38900	27.73	0.54265	
